# Mapping Evidence in Teaching Palliative Care in Undergraduate Curriculum of Healthcare Professionals Qualification: A Systematic Scoping Review Protocol

**DOI:** 10.21203/rs.3.rs-4313706/v1

**Published:** 2024-07-03

**Authors:** Sphamandla Josias Nkambule, Bernhard Gaede

**Affiliations:** University of KwaZulu-Natal School of Nursing and Public Health; University of KwaZulu-Natal Nelson R Mandela School of Medicine: University of KwaZulu-Natal College of Health Sciences

**Keywords:** Palliative Care, End-Of-Life Care, Terminal Care, Education, Primary Health Care, Training, Healthcare Professionals

## Abstract

**Background:**

Clinical Practice Guidelines recommend interprofessional collaboration in palliative care. However, healthcare profession educators lack clear curricular guidance, particularly for undergraduate programs, to adequately train future professionals for effective participation in such teams.

**Objective:**

This systematic scoping review protocol aims to address this gap by: (i) mapping evidence on key characteristics of teaching palliative and end-of-life (EOL) care to undergraduate healthcare and social care students, and (ii) identifying the nature and effectiveness of educational interventions for improving palliative care education in the undergraduate curriculum.

**Methods and Expected Outputs:**

The protocol adheres to the Preferred Reporting Items for Systematic Reviews and Meta-Analyses Protocol Extension for Scoping Reviews (PRISMA-P-ScR) guidelines, while the proposed systematic scoping review study will be conducted based on methods and steps identified by Arksey and O’Malley and experts in the field. We will conduct systematic searches across five EBSCOhost databases using relevant search terms. Additionally, a limited grey literature search will be conducted on the first 100 results of Google Scholar and Open-Grey. The selection process will follow PRISMA-Extension for Scoping Reviews. Two independent reviewers will screen titles and abstracts for eligibility. Data extraction will be done on standardized forms in duplicate with cross-checking by a third reviewer. Braun and Clarke’s thematic analysis approach, combining thematic and directed content analysis, will be employed for analysis. Intervention effectiveness will be narratively summarized based on the TIDieR checklist. Meta-analysis will be considered if applicable and data is homogeneous.

**Discussion:**

Palliative care education (PCE) is a crucial element of undergraduate health professions education. This study’s findings may aid educators in fostering optimal learning among healthcare students, who can then positively influence community health outcomes.

**Ethics and dissemination:**

The protocol was submitted for ethical clearance to the University of KwaZulu-Natal’s Biomedical Research Ethics Committee and granted exemption from ethics review (00024289). We will disseminate findings through scientific journal publication and by sharing a summary with relevant institutions and attendees at health promotion and interprofessional education conferences.

## STUDY BACKGROUND

A staggering 78 per cent of the estimated 56.8 million people who need palliative care globally reside in low- and middle-income countries (LMICs), including 25.7 million in the last year of life. This translates to only 14 per cent of those who could benefit from palliative care actually end up receiving it, highlighting a significant gap in access, particularly for LMIC populations.^1^ Remission and survival rates for palliative care patients in most African countries are adversely affected by late detection, diagnosis, limited training of health care providers, and lack of resources for treatment and palliative care services in health facilities.^2,3^

South Africa’s unique disease burden, characterised by a high prevalence of HIV, tuberculosis (TB), and non-communicable diseases (NCDs) like cancers and injuries, intensifies the existing gap in palliative care services. This is particularly true for infectious diseases like TB and HIV, which are known to cause significant suffering in LMICs.^4^ This underscores the critical necessity for comprehensive and integrated approaches to palliative and end-of-life care education and service provision, especially in regions facing such significant health challenges.

### Health Policy Context of the study: Global, Continental and National

The pressing need for palliative care in Africa is further highlighted by the World Health Assembly Resolution 67.19 passed in 2014. This resolution specifically called on member states to integrate palliative care into comprehensive care throughout a person’s life.^5^ Request number four of the resolution explicitly addresses education and training, urging countries to include palliative care as a standard part of healthcare professional education and training. This includes integrating basic and continuing education on palliative care into undergraduate medical and nursing programs, as well as in-service training for care providers at the primary care level, including doctors, nurses, primary care workers (such as community health workers), spiritual caregivers (those who provide emotional and religious support to patients and families), and social workers.

However, despite international recognition and clear steps outlined in resolutions like the World Health Assembly Resolution 67.19, translating the definition of palliative care into accessible services for Africans remains a challenge. This disconnect between the urgent demand for palliative care in regions such as South Africa and the insufficient advancements made in establishing comprehensive educational and training initiatives, as advocated by international resolutions underscores the need for stronger implementation efforts to ensure that palliative care becomes a true pillar of Universal Health Coverage (UHC) on the continent.

Countries can not meet the United Nations Sustainable Development Goal Three, specifically Target 3.8, without including palliative care and pain relief.^6^ South Africa has adopted the policy of National Health Insurance intended to promote access to promotive, preventive, curative, rehabilitative and palliative care services of sufficient quality to be effective while not exposing citizens to financial hardship.^7^

### Palliative Care in Undergraduate Health Professions Education.

A scoping review was conducted to examine the provision of Palliative Care services in Africa from 2005 to 2016.^4^ The study followed the guidelines set by the World Health Organisation (WHO) for the public health strategy of palliative care,^8^ which include the implementation of services, educational initiatives, policy development, medicine availability, and professional engagement. The review findings revealed a significant presence of these services in Kenya, South Africa, and Uganda.^4^ Stand-alone palliative care policies exist in Malawi, Mozambique, Rwanda, Swaziland, Tanzania, and Zimbabwe.^4^

The South African National Department of Health adopted the National Policy Framework and Strategy on Palliative Care 2017–2022 to promote the development and delivery of Palliative Care services.^7^ However, despite these efforts, the lack of access to Palliative Care services on the African continent was further emphasised by the Lancet Commission Report, which provided recommendations concerning Human Resources in this context: “Establish palliative care as a recognised medical and nursing speciality; Make general palliative care and pain relief competencies a mandatory component of all medicine, nursing, psychology, social work, and pharmacy undergraduate curricula; Require that all health and other professionals involved in caring for patients with serious, complex, or life-threatening health conditions receive basic training in palliative care and pain relief.”^3^

Current undergraduate health professional curricula throughout Africa have elements of Palliative Care education that are poorly quantified despite palliative care being recognised as an emerging discipline in health care. Therefore, there is an urgent need to review and strengthen curricula to provide an inter-professional approach to palliative care education for the African health professional, allowing future fit professionals to competently deliver on the growing Palliative Care needs of the continent.

This will lay the foundation for a sustainable, high-quality and accessible palliative care system integrated into primary health care, community and home-based care, and supporting care providers such as family and community volunteers.^9^

### The rationale for the study

While accreditation bodies have mandated teaching basic palliative and end-of-life (EOL) care across healthcare professions training curricula,^9^ there has been limited guidance for educators on what or how to teach and evaluate students’ competence, particularly in the undergraduate curriculum for HCP qualification. Moreover, there is a lack of extensive evidence regarding the nature and effectiveness of educational interventions to improve palliative care education and whether these interventions result in positive outcomes.

A preliminary systematic search was performed using the EBSCOhost platform, searching two databases, Medline and CINAHL, and finding no scoping reviews for palliative care education and training in the undergraduate curriculum for HCP qualification. In addition, the two international perspective registers of systematic and scoping review databases, PROSPERO and the Open Science Framework (OSF), were searched respectively, and no similar review protocols were identified. Therefore, due to this limited evidence, our aims were broad, which was more suited for a scoping review rather than a systematic review, which would be, to sum up, the best available research on palliative care education (PCE) across the undergraduate curriculum for HCP qualification.

### Aim

This scoping review aims to systematically map the research done in palliative care education (PCE), as well as to identify the nature and effectiveness of educational interventions to improve palliative and end-of-life (EOL) care education across the undergraduate curriculum for HCPs qualifications.

### Objectives

To systematically map the scope of available published knowledge about teaching palliative and EOL care across the undergraduate curriculum for HCP qualification.

### Sub-objectives

1. To identify studies that have used educational and training interventions for palliative and end-of-life (EOL) care teaching across HCP training curricula;
2. To critically appraise and identify curricular components of effective interventions in the undergraduate curriculum for HCP qualification; and
3. To use this critically appraised evidence base to inform the design of palliative and end-of-life (EOL) care teaching across HCP training curricula.

## METHODOLOGY

### Study Design - Protocol and registration

This scoping review protocol is part of a more extensive **study [Details of the bigger project goes here**], and the protocol for the complete review is available in the PROSPERO data repository registries – ID CRD42023481892 https://www.crd.york.ac.uk/prospero/export_details_pdf.php.

As part of the more extensive study, a scoping review was chosen as the best method to systematically synthesise qualitative and quantitative evidence on the nature and effectiveness of currently available educational interventions to improve palliative care education for health and social care professionals in the undergraduate curriculum. The protocol for the scoping review was developed per the Preferred Reporting Items for Systematic Reviews and Meta-Analyses Protocol extension for a scoping review (PRISMA-P-ScR), which permitted the forwarding of a six-step scoping review protocol (see Supplementary Appendix for the completed PRISMA-P Checklist).^10^

**Figure 1:**
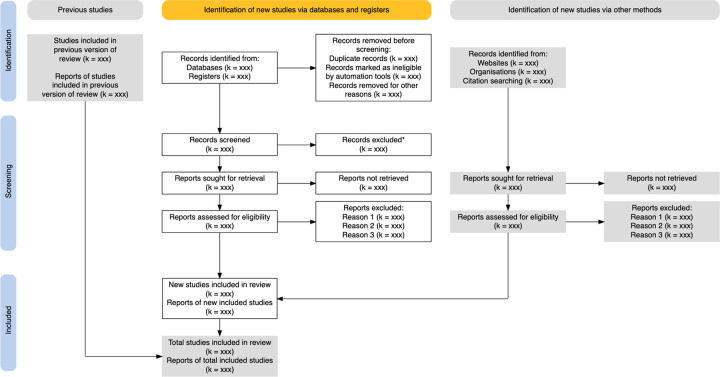
Preferred Reporting Items for Systematic Reviews and Meta-Analysis (PRISMA) 2020 flow diagram will be used to report the study selection and eligibility screening process results | Source: Adopted from Page, Matthew J., et al.13

The proposed comprehensive systematic scoping review of the literature will be conducted according to the Levac *et al*. (2010) adaptation to Arksey and O’Malley (2005) framework for scoping review methods and experts in the field.^11^, ^12^ The review findings will be reported per the PRISMA 2020 statement - an updated procedure for reporting review studies (see Fig. 1 for the PRISMA-2020 flowchart).^13^

### Step 1: Identifying the review question

As an integral component of the framework’s preliminary phase, the research team received guidance from the expert team in formulating comprehensive and specific research questions. Additionally, the study protocol’s design, the selection of search terms for the literature review, and the identification of relevant databases were established through a collaborative process involving iterative consultations with our research team, key informants possessing expertise in curricular design, implementation, and assessment of healthcare professional qualifications in both general and specific contexts related to palliative and end-of-life care. Furthermore, the input of an experienced medical librarian was sought in this process.

**Figure 2:**
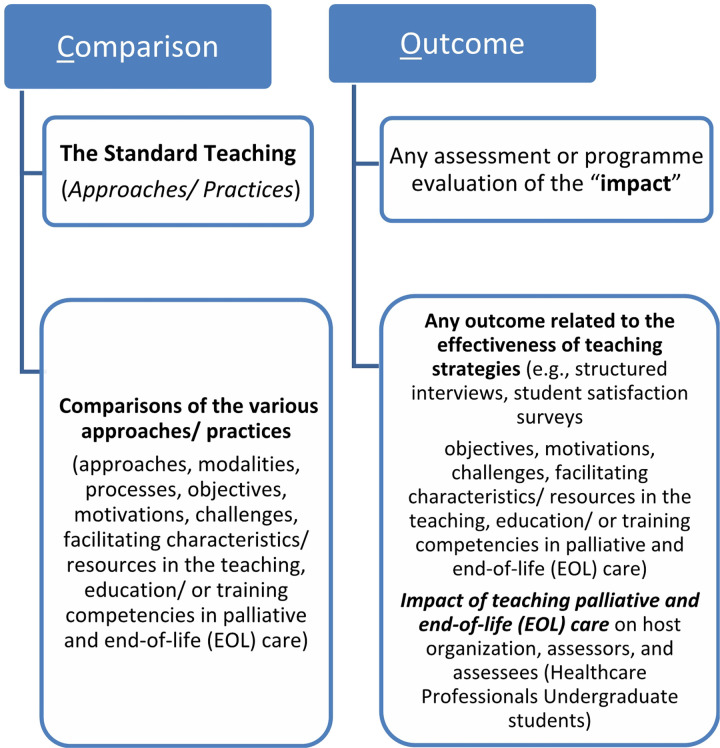
Framework for determining the eligibility of the research questions | Source: The first and second authors designed and developed the diagram in accordance with the PICO nomenclature framework recommended by the JBI Manual for Evidence Synthesis.14

To determine whether the research question qualified for a scoping review project, we utilised the PICO (participants; interventions; comparator; outcomes) nomenclature framework recommended by the Joanna Briggs Institute (JBI) Manual for Evidence Synthesis: 2020 Edition (Fig. 2).^14^

The main research question that the proposed scoping review will address:

What is the nature and effectiveness of teaching palliative and end-of-life (EOL) care to students in the undergraduate health and social care professionals qualifications curriculum?

More specifically, the scoping review seeks to address the following research sub-questions:

1. What is the evidence on the nature of educational interventions to improve palliative and end-of-life (EOL) care teaching in the undergraduate curriculum for health and social care professionals qualifications?
2. What is the effectiveness of educational interventions to improve palliative and end-of-life (EOL) care teaching in the undergraduate curriculum for health and social care professionals qualifications?

The eligibility criteria for including studies in this review in order to answer the aforementioned questions are based on the relevant components of the PICOd-T (Study Design - Time) framework and the research question. Thus, the PICOd-T framework is employed to ensure that the boundaries of the proposed scoping review research question are clearly defined.

The eligible studies will be included once they have been evaluated independently, reproducibly, and systematically by two reviewers. For studies to be considered eligible, studies must provide evidence of one of the factors listed in Table 3. A third reviewer will resolve disagreements between the two reviewers; reviewers will also pilot-test the inclusion/exclusion criteria to reach a consensus before beginning the study selection process (**Step 3: Study selection and eligibility screening**).

### Step 2: Identifying relevant studies - Search Strategy and Data Sources

The systematic, comprehensive, and reproducible searches to identify relevant studies will initially be conducted via electronic search sources – searching reputable bibliographic databases and indexing services (and platforms) – followed by searches of supplementary information sources to capture primary studies addressing the main review question. With the assistance of a professional medical librarian, the first author will conduct all direct electronic and supplementary information source systematic searches, using a pre-defined and piloted search strategy (see Table 1), to capture both published and unpublished (grey) literature to be screened for eligibility for inclusion in this review.

### Search Strategy

The first author, a medical librarian and subject specialist with experience in designing, implementing, and assessing HCP qualifications in general and in the palliative and EOL care domain, co-developed the comprehensive search strategy (see Table 1 for details of the search strategy metrics). All authors were given an equal opportunity to review the draft to ensure the correct use of indexing terminology and Medical Subject Headings (MeSH) descriptors before it was pilot-tested on a subset of records from the PubMed database (see Table 1 pilot test results).

In accordance with the approach of Bethel *et al*., (2021), a search summary table (SST) will be used to report on the performance of the search strategy in effective searching databases. The SST is a viable and reproducible method for reporting and evaluating the search strategy’s effectiveness (see Table 2 for the example of the SST metrics that will be used for this purpose).^15^

### Data Sources

#### Electronic search sources

The systematic and comprehensive searches will be conducted from the following electronic databases to source articles published on the topic of teaching palliative and end-of-life (EOL) care to students in the undergraduate curriculum of HCPs qualification, using the pilot-tested search strategy:

WEB of Science, PubMed, and EBSCOHost Web (Academic Search Complete, PyscInfo, MEDLINE with Full Text, and Health Source: (Nursing/Academic Edition)). Furthermore, evidence on palliative care teaching interventions will be sourced from the South African National Clinical Trial Register and the ISRCTN registry. The mentioned databases will be searched from their inception to the present, regardless of the publication language. This procedure aims to obtain as many articles as possible from electronic search sources and to ensure that all relevant articles or reports are captured before the study selection and eligibility screening process begins.

#### Searching other resources – supplementary information search

In addition, supplementary search methods will include hand-searching of relevant journals, reference lists of identified peer-reviewed articles and grey literature, and as well as forward and backwards citation chasing. The reviewers will further browse through the link entitled “Related Articles” option, which searches for similar citations using an intricate algorithm that scans titles, abstracts, and MeSH terms to detect more studies (Table 3).

Systematic reviews and other review papers are not eligible for inclusion; however, reference lists of relevant reviews, preprints, and conference abstracts will be screened for more relevant primary studies not captured by the search strategy. Furthermore, the appropriate trial publications reference lists will be checked for unidentified randomised clinical trials.

#### The systematic search management

The SST will also be used to present and keep records of the systematic searches retrieved information, such as the summative metrics of effective searching suggested by Cooper *et al*., (2018), and additional metrics providing further useful search-related information for the librarian or information specialist.^16^

The SST will be completed in two stages. In stage one, all the references that the search strategy retrieves from each electronic database, including all duplicates, will be exported to EndNote X9 (version 19.1.0.12691) – a reference management software, which will be used to create a virtual library (Thomson Reuters, Stamford, CT, USA).^17^ Every record in the virtual library will be given a code for the database name where the record was found.

Stage two involves re-running the searches in the databases where most of the included references were found to determine whether references not found during the original search were in the database and, if so, whether the search strategy retrieved them.

#### Step 3: Study selection and eligibility screening

The study selection and eligibility screening method is multi-step and involves two reviewers. The eligibility screening approach for eligible articles will be carried out in accordance with the Preferred Reporting Items for Systematic Reviews and Meta-Analysis (PRISMA) criteria, as represented in the PRISMA 2020 Flow Diagram Fig. 1. First, two reviewers will screen the title and abstracts for relevance independently and in duplicate, and articles that pass initial screening by either reviewer will undergo full-text review independently and in duplicate by the same two reviewers. Standardised, pilot-tested eligibility forms will be used for both title and abstract screening and for full-text review (see Supplementary Appendix for the completed screening tool).

Disagreements about study eligibility will be resolved through consensus discussion or will be resolved by a third reviewer in the case of ongoing disagreement. Kappa statistics will be calculated to assess the inter-rater reliability of full-text review, using Cohen’s Kappa coefficient (κ) statistic on Stata 13.0SE (StataCorp College Station, TX, USA), a robust statistic used for inter-rater reliability testing.^18^

### Step 4: Data extraction/ or collection process

A standardised data extraction form informed by the template for intervention description and replication (TIDieR) checklist and guide for describing interventions will be created to capture relevant information from the included studies (see Supplementary Appendix for the data extraction form).^19^ The form will consist of fields for study characteristics, participant demographics, teaching/ or communication strategies employed, outcome measures, and key findings

Two independent reviewers will extract data from the included studies using the standardised data extraction form to detect inter-rater errors and decrease data errors and bias. The reviewers will extract data from the included articles independently and in duplicate. A third reviewer will verify the data related to study characteristics, such as publication information, study dates, population characteristics, interventions, outcomes, and study methods, which are required to assess the risk of bias.

In the meta-analysis, the nature of the data, such as continuous outcomes (mean differences or standardised mean differences) or dichotomous outcomes (odds ratios or risk ratios (ORs)) will be calculated if necessary, and if the data is available, only measures most adjusted for one or more sets of potential confounders such as socio-demographic and lifestyle factors will be extracted by the two reviewers to reduce confounding and measurement errors and to ensure consistency across studies and reduce bias.

#### Requests for missing data

Missing data will be identified and recorded within the review. Where papers provide insufficient details about the intervention, such as what is delivered and by whom, an effort would be made to contact the authors to obtain previously unpublished information and clarify any missing data. In cases of non-response or inadequate clarity from contacted authors (i.e. retrieval of missing data is not possible), that study/outcome will be eliminated from the review.

### Step 5: Assessment of study methodological quality, risk of bias, and the certainty of evidence

The methodological quality and risk of bias of each included study will be assessed by two reviewers independently, using appropriate methodological quality assessment tools depending on the design of the included studies using the guidance produced by the NHS Centre for Reviews and Dissemination and the Cochrane Collaboration.^20, 21^

#### Tools to assess the methodological quality

As recommended, the MERSQI (Medical Education Research Study Quality Instrument) Scale and Newcastle-Ottawa Scale Education (NOS-E) [19, 20] will be used to determine the methodological quality of the included studies.^22^, ^23^ The two instruments will be used as they assess different aspects of quality and risk of bias, acting in a complementary fashion. As described in previous studies, a score above the sample median MERSQI score (12.5) and NOS-E score (2.5) will be considered the threshold for high methodological quality.^22^

Furthermore, the risk of bias for RCTs will be assessed using the Cochrane risk of bias tool, which will take into account random sequence generation, allocation concealment, participant and personnel blinding, insufficient outcome data, and selective reporting. Each domain will be evaluated independently by both reviewers and classified as ‘high,’ ‘low,’ or ‘uncertain’ regarding bias. Studies will be considered to be at an overall ‘high’ risk of bias if they are judged to be at ‘high’ risk of bias in any domain, ‘uncertain’ risk of bias if they are judged to be at an uncertain risk of bias in any one domain, with no domains at high risk of bias, and an overall ‘low’ risk of bias if they are not judged to be at ‘high’ or ‘uncertain’ risk of bias in any domain. All risk of bias assessment will be judged at the outcome level.

#### Tools to assess the risk of bias (or publication bias)

Publication bias will be assessed using visual inspection of funnel plots, where sufficient numbers of studies will exist to permit interpretation.^24^

##### Tools to assess the certainty of evidence (quality of evidence/ the strength of the body of evidence)

The overall quality of evidence (or certainty in the findings) for each outcome obtained will be evaluated using the five GRADE principles (trial bias risk, consistency of effect, imprecision, indirectness, and publication bias). The completed GRADE checklist and reasons for up- or down-grading assess the quality of a body of evidence based on study methodological quality, results from sensitivity analysis, and by downgrading and upgrading the baseline quality score according to the domains specified in the GRADE guidelines.^25^ We will generate a table that summarises our findings using GradePRO software.^26^

### Step 7: Collating, summarising, and reporting the results

Systematic scoping reviews provide an executive overview of current evidence by answering broad questions.^27^ Thus, following the completion of the data extraction step, we will generate a comprehensive summary of the data extracted from the included studies. The summary will consist of two key presentation components:

1. **Numerical presentation**: We will create a compiled findings table that provides a descriptive overview of the key pre-specified study outcomes and type of included studies, as depicted in Fig. 2. Additionally, we will employ an interactive web-based graphic design tool, specifically Canva version 2.93.0, to generate a world map.^28^ This map will help visualise the geographical distribution of the summary of the included studies, offering insight into global patterns.
2. **Narrative presentation:** To ensure a holistic analysis, we will synthesise the essential characteristics of all the included studies. We will archive this by documenting the structure of the intervention of interest – all educational interventions used to teach palliative and end-of-life (EOL) care education to health and social care professionals in the undergraduate curriculum – will be captured by recording pedagogy, course content and learning outcomes. This will consist of data including, but not limited to, educational method, educational content, length/duration, outcome measures, outcome results and implementation ‘barriers and facilitators’ such as financial support.

#### Strategy for data synthesis

The data synthesis process will involve the following steps:

1. **Thematic Analysis**: We will conduct a thematic analysis of the extracted data to identify recurring themes, concepts, and patterns related to key characteristics of teaching palliative and end-of-life (EOL) care to students in the undergraduate health and social care professionals qualification curriculum. This will involve coding and categorising the extracted data into meaningful themes and sub-themes;
2. **Qualitative Summary**: We will provide a qualitative summary of the key findings from the included studies. This will involve synthesising the results narratively, highlighting similarities, differences, and trends in the effectiveness of a series of identified interventions to improve palliative care education for health and social care professionals in the undergraduate curriculum;
3. **Subgroup Analysis:** If a sufficient number of studies with similar characteristics and outcomes are identified, we may conduct subgroup analyses based on factors such as study design, geographical location, or types of Educational methods/ Pedagogies employed in the Teaching of PC. This will enable us to explore potential variations in the effectiveness of different interventions – to improve palliative care education for health and social care professionals in the undergraduate curriculum – within specific subgroups; and
4. **Meta-analysis (if applicable):** If the included studies are deemed suitable and homogeneous, we will consider conductinga meta-analysis to provide a quantitative synthesis of the data.

Furthermore, the authors will delve into the implications of these findings for future research, practice, and policy.^12^ Stage 7 details are still being worked out, and they may be repeated or changed if reviewers deem them necessary.

#### Ethics and dissemination

No ethical clearance is required for this study. The results of the proposed systemic scoping review will be disseminated electronically, in print, and through conference presentations as well as key stakeholder meetings.

## DISCUSSION

The proposed systematic scoping review aims to deliver comprehensive evidence on palliative care education (PCE) and identify the nature and effectiveness of educational interventions to teach palliative and end-of-life (EOL) care education to health and social care professionals in the undergraduate curriculum. Most importantly, the proposed scoping review’s purpose is not to rate the quality of evidence or come to conclusions about best practices, but rather to offer the scope of available published knowledge about teaching palliative and EOL care across the undergraduate curriculum for health and social care professionals qualification.

We believe that by adopting this approach, we will be able to ascertain the educational experience of both learners and educators. This will entail taking into account the needs of learners, the interactions between learners and educators, the interactions among learners themselves, the contributions made by the department, and the utilisation of information to nudge behaviours and improve decision-making skills around palliative and EOL care. Furthermore, a key area of work that will commence as part of the project’s more extensive study (and is likely to outlive the lifespan of the project) is to engage with key stakeholders regarding the inclusion of palliative and EOL care into all undergraduate curricula for health and social care professionals qualification programs in Africa. It is envisaged that a consensus statement will be developed and shared in a range of forums, in particular the African Palliative Care Association, Africa-wide family medicine structures such as Primafamed and WONCA Africa as well as AFREhealth, in which they will be consulted, thus providing valuable insights beyond what has been captured through scoping review project.

### Strengths and limitations of this study

A strength of this study is that it will apply a broad review of multidisciplinary databases covering Public Environmental Occupational Health, Nursing, Health Care Sciences Services, Medicine General Internal, Education Scientific Disciplines, Educational Research, Social Sciences Interdisciplinary and other Web of Science Core Collection Citation Topic providing a comprehensive assessment of published literature on courses designed on these subjects. See the Supplementary Appendix file for the search strategy pilot results analysis completed for the Web of Science database report.

A limitation of this study is that only peer-reviewed literature in English will be included, which will limit the scope of this review to articles published in English-speaking countries or those published in English.

## Figures and Tables

**Table 1:** Search strategies with MeSH descriptors and truncation, with pilot-test results from PubMed, will be adapted for other databases.

Number	Search Terms	Record Retrieved
# 1	**Health Professions Undergraduate Students:** Medical Doctors OR Nursing Professionals OR Midwifery Professionals OR Midwives OR Dentists OR “Dieti*” OR “medical technology*” OR Pharmacists OR Dentist OR dental assistant OR emergency care practitioner OR emergency care assistant OR emergency care technician OR Emergency Medical Technician OR Occupational Therapist OR Physiotherapy Therapist OR Dialysis Technician OR Speech Language Pathologist OR Audiologist OR Physiotherapy OR Optometrist OR Healthcare Chaplain OR General practitioner OR Primary care physician OR Community Health practitioner OR Pharmacy technician OR Dental Therapy OR Oral Hygiene OR Epidemiologist OR Public Health Nurse OR Radiologist OR Clinical Technology OR health profession* OR “Healthcare profession*”AND	82,597
# 2	**Palliative and End-of-Life (EOL) Care:** “palliative care” OR “dying with dignity” OR “pain management” OR hospice* OR grief* Or bereavement* OR Terminal care” OR Hospic* OR “Hospice Care” Or “palliative care education” OR education* AND	186,448
# 3	**Education, Teaching:** “educational programs” OR programs* OR Student OR Undergraduate OR seminars* OR classes* Or teaching* OR teach OR educat* OR training* OR Education OR Training OR Learning OR Teaching OR Workshop* AND	615,583
# 4	**Combined Systemic Searches:** # 1 AND # 2 AND # 3	3,448

**Note:** The Comparators (*The Standard Teaching*) and Outcomes (*Any assessment of the “impact.”*) is as reported in the study. **Free text terms / natural language terms:** (synonyms, UK/ US terminology, medical/ laymen’s terms, acronyms/abbreviations, drug brands, more narrow search terms) *Consider: phrase searching, proximity operators, truncation, wildcards, field qualification (e.g. textword)*. **Controlled vocabulary terms / Subject terms** (MeSH terms, Emtree terms) *Consider: explode, major headings, subheadings*

**Table 2:** The Search Summary Table (SST), will be used to report on the search strategy performance.

Project Title: Teaching Palliative Care in the Undergraduate Curriculum of Healthcare Professional Qualification: A Systematic Scoping Review											
Included references	Format	Database Searches [ *date run: / / ; date re-run:* / / ]					Supplementary Searches				
		PubMed	Web of Science	PsycINFO	ClNAHL	MEDLINE	fcs	bcs	hs	wss	org
Included ref 1											
Included ref 2											
No. of included refs											
No. of unique refs											
Yield		3,448									
No. of refs screened											
Sensitivity											
Precision											

Search-related information for the librarian or information specialist	The summative metrics of effective searching
No. of database searched =	Overall sensitivity =
Sum of yields =	Overall precision =
No. of refs that underwent Title & Abstract screening	NNR =
No. of refs that underwent Full-text (FT) screening =	NNR FT =
No. of included refs from database searching =	NNS =
Total no. of included refs =	

Codes	Supplementary search codes	Format codes
x = found from the search	fcs = forwards citation search	jnl = journal article
y = in database; found when search strategy re-run	bcs = backwards citation search	ths = PhD thesis
n = not in the database	hs = hand search	
z = in the database; not found using the search strategy	wss = web site search	Other codes
(red) = databases where searches re-run	org = from contacting organisations	FT = full text
NNR = number needed to read. 1/overall precision		
NNR FT = number needed to read at FT to find one included reference		
NNS = number needed to screen to find one reference to include for FT screening		

**Table 3:** Eligibility criteria according to the PICOd-T (Study Design - Time) nomenclature framework

Criteria	Inclusions	Exclusions
**Population**	Studies conducted in any part of the world with human participants, Healthcare Professions Undergraduate students/ or graduate-entry programmes students and/or academic staff teaching across health profession, both fulltime and part-time students within the clinical, medical, research and/ or educational setting, irrespective of study setting, will be eligible for inclusion. Reported study participants will be included irrespective of gender, ethnicity, culture, race, and comorbidities background.	Research studies involving nonhuman participants as participants, Non-HCPs Undergraduate students.
**Intervention (or Exposure)**	Studies with human participants (health professional students) exposed to formal palliative and End-of-Life (EOL) care teaching/ or curriculum content and describe teaching methods, approaches/ or practices employed with a focus on achieving stated learning outcomes/ objectives and associated curriculum evaluation included. Studies will be included either reporting on intervention pedagogies in teaching palliative and end-of-life (EOL) care. For example: Focused on Reflection, Experiential Learning, Role-modelling, Dialectic Methods, and Didactic Methods	Studies that focused on ad-hoc palliative/ or EOL care learning experiences (e.g., a once-off visit to an EOL unit), rather than integrated course content Studies which do not adequately describe the intervention will be excluded.
**Comparator**	Studies reporting on outcomes of interests, interventions support with no support/ or standard teaching (approaches/ practices) will represent the comparator. Comparisons of the various approaches/ practices For example: modalities, processes, objectives, motivations, challenges, facilitating characteristics/ resources in the teaching, education/ or training competencies in palliative and end-of-life (EOL) care)	
**Outcomes**	Studies reporting on any outcome related to the effectiveness of teaching strategies (e.g., structured interviews with students, student satisfaction surveys, tests and exams, content analysis, peer observation feedback or specific outcome measures) in palliative and end-of-life (EOL) care) For example impact of teaching palliative and end-of-life (EOL) care on the host organisation, assessors, and assessees (Healthcare Professionals Undergraduate students), any assessment or programme evaluation of the “impact”; “outcome*”; “benefit”; AND the achievement of “attributes”; “skill*”; “knowledge”; “behaviour”; “personal growth” or “reflect*”; “transformation.”	Research studies reporting on other outcomes, not in line with the outcomes of interest. Studies that did not report clear outcomes
**Study Design**	Only original, primary studies of both quantitative and/ or qualitative study designs that are peer-reviewed and published in English, irrespective of publication year, will be eligible for inclusion	Reviews studies (However, reference lists of relevant reviews will be checked for primary studies) Authors of studies published only as abstracts will be contacted and asked to provide further detail. If no further detail is available, the study will be excluded.
**Time**	From inception – up to April 2023	
**Language**	Studies written in English will be included to avoid loss or distortion by translation from studies written in another language.	Non-English language quantitative and qualitative studies will be translated.

Note: Initial exclusion criteria were studies published in languages other than English and did not have the search keywords in the title or abstract. The inclusion/ exclusion criteria will be tested through piloting by two reviewers

## Abbreviations

EOL: care End-of-life
WHO: World Health Organization
PRISMA-P-ScR: Preferred Reporting Items for Systematic Reviews and Meta-Analyses Protocol Extension for Scoping Reviews
HCPs: Healthcare Professionals
